# An intact animal model for the assessment of coronary blood flow regulation “Coronary blood flow regulation”

**DOI:** 10.14814/phy2.14510

**Published:** 2020-07-30

**Authors:** Céline Boudart, Fuhong Su, Antoine Herpain, Jacques Creteur, Robert Naeije, Serge Brimioulle, Laurence Dewachter, Luc Van Obbergh

**Affiliations:** ^1^ Department of Anesthesiology Erasme University Hospital Université Libre de Bruxelles Brussels Belgium; ^2^ Department of Intensive Care Erasme University Hospital Université Libre de Bruxelles Brussels Belgium; ^3^ Laboratory of Physiology and Pharmacology Faculty of Medicine Université Libre de Bruxelles Brussels Belgium

**Keywords:** autoregulation, coronary blood flow, endothelial function, metabolic regulation, microvascular function

## Abstract

Coronary blood flow adapts to metabolic demand ("metabolic regulation") and remains relatively constant over a range of pressure changes ("autoregulation"). Coronary metabolic regulation and autoregulation are usually studied separately. We developed an intact animal experimental model to explore both regulatory mechanisms of coronary blood flow. Coronary pressure and flow‐velocities were measured in four anesthetized and closed‐chest pigs using an intracoronary Doppler wire. Metabolic regulation was assessed by coronary flow reserve defined as the ratio between the maximally vasodilated and the basal flow, with hyperemia achieved using intracoronary administration of adenosine (90 µg) or bradykinin (10^–6^ M) as endothelium‐independent and ‐dependent vasodilators respectively. For both vasodilators, we found a healthy coronary flow reserve ≥ 3.0 at baseline, which was maintained at 2.9 ± 0.2 after a 6‐hr period. Autoregulation was assessed by the lower breakpoint of coronary pressure‐flow relationships, with gradual decrease in coronary pressure through the inflation of an intracoronary balloon. We found a lower limit of autoregulation between 42 and 55 mmHg, which was stable during a 6‐hr period. We conclude that this intact animal model is adequate for the study of pharmacological interventions on the coronary circulation in health and disease, and as such suitable for preclinical drug studies.

## Graphical abstract

Metabolic regulation adapts the coronary blood flow (CBF) to the myocardial demand. Autoregulation maintains CBF constant over a wide range of pressure. These CBF regulation mechanisms were to date studied separately. We developed here a new method to comprehensively assess CBF regulation in an intact large animal experimental model.

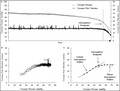

## 1 INTRODUCTION

Cardiac muscle is characterized by a high oxygen (O_2_) uptake along with a high O_2_ extraction with limited anaerobic capacity (Tune, Gorman, & Feigl, 2004). Without adequate O_2_ delivery, there is rapid onset of myocardial ischemia and decline in contractility. CBF is determined by autonomic nervous system tone and metabolic demand (i.e. "metabolic regulation") (Feigl, Neat, & Huang, 1990; Tune et al., 2004) and remains constant over a wide range of pressures (i.e. "autoregulation") (Canty, 1988; Dole, 1987). CBF may increase by up to three times by metabolic regulation. This maximal increase in CBF is known as CBF reserve (Kern et al., 2006). On the other hand, autoregulation of CBF fails when coronary perfusion pressure drops below a critical value. At that point, CBF becomes directly dependent on perfusion pressure (Duncker, Koller, Merkus, & Canty, 2015; Hoffman & Spaan, 1990). Autoregulation of CBF occurs mainly in the microcirculation, which contributes for more than 90% of the coronary vascular resistance (Chilian, 1997). In response to physiological triggers, such as shear stress or metabolic changes, the vascular endothelium regulates arterial smooth muscle tone through a fine balance between vasodilators and vasoconstrictors release. Microvascular and endothelial function therefore play a key role in CBF regulation (Durand & Gutterman, 2013; Furchgott & Zawadzki, 1980).

To date, each of these regulatory mechanisms have been studied separately. We thought that a comprehensive method to assess integrated CBF regulation might be of interest. Therefore, the aim of the present research work was to develop a valid, stable, and reproducible method to assess, in a unique methodological protocol, the metabolic regulation – including the microvascular and endothelial functions – and the autoregulation, especially its lower autoregulatory breakpoint. An intracoronary Doppler wire was used to measure coronary pressure and flow velocities. An intact porcine model was implemented to ensure a maximum translational pertinence of the obtained results.

## 2 MATERIALS AND METHODS

The present study was approved by the Institutional Ethics Committee on Animal Welfare from the Faculty of Medicine from the *Université Libre de Bruxelles* (Brussels, Belgium; acceptation number: 584N). Animal care and handling were in accordance with National and Institutional Guidelines for the care and use of animals.

### 2.1 Animals handling

#### 2.1.1 Premedication and Preparation

Four healthy domestic 4‐month‐old pigs were included in the study. Pigs were fasted for 12 hr prior the start of the experiment with free access to water. To prevent unnecessary stress and discomfort, the pigs were premedicated with intramuscular midazolam (50 mg/kg), azaperone (200 mg), and atropine (1mg) fifteen minutes before their transport to the operating room. After placing the animal in the supine position on the operating table, a five leads electrocardiogram was used to monitor heart rate and a 14G peripheral catheter (Terumo® Corporation, Leuven, Belgium) was inserted in the ear vein for intravascular access. A femoral artery catheter (Vygon®, Brussels, Belgium) inserted under ultrasound guidance monitored the arterial blood pressure and allowed serial blood sampling.

#### 2.1.2 Anesthesia, ventilation, and fluid filling

The animals were anesthetized with intravenous propofol 2% (1–2 mg/kg) and sufentanil (2–3 µg/kg). Balanced anesthesia was maintained and titrated with inhaled sevoflurane (1%–2%) and continuous intravenous sufentanil (4–10 µg kg^−1^hr^−1^) until the end of the procedure. Adequate analgesia and anesthesia were defined as the absence of clinical and hemodynamic manifestations to a painful pinching of snout test. Muscle relaxation was provided by intravenous rocuronium (1.2 mg/kg bolus for induction and 2 mg kg^‐1^hr^‐1^ continuous intravenous infusion for maintenance). After oro‐tracheal intubation with a 8.0 mm diameter cuffed endotracheal tube, mechanical positive pressure ventilation was initiated with continuous capnography (tidal volume of 10 ml/kg; respiratory rate of 14 breaths/min, positive end‐expiratory pressure of 5 cm H_2_O and a fraction of inspired oxygen of 0.3). The ventilator settings were permanently adjusted to maintain a PaO_2_ of 90–100 mmHg, a PaCO_2_ of 35–45 mmHg, and a peak airway pressure below 30 cm H_2_O. Intravenous infusion of a crystalloid solution (PlasmaLyte®, Baxter SA, Lessine, Belgium) was administered at 10 ml kg^‐1^ hr^‐1^ rate during instrumentation and surgical procedure and reduced to 7 ml kg^‐1^ hr^‐1^ rate afterwards. Normoglycemia (arterial blood glucose levels of 80–120 mg/ml) was maintained throughout the whole experiment using 20% glucose infusion as required. Body temperature was kept constant (37–39°C) with heating pads or cooling.

#### 2.1.3 General Instrumentation and Surgical Preparation

Neck, hind limb area, and abdomen were cleaned and disinfected with iodine 2%. Under ultrasound control and using the Seldinger technique, the right common carotid artery and the remaining femoral artery were cannulated using a 6‐F introducer (Terumo® Corporation, Brussels, Belgium). The right external jugular vein was cannulated using a 8.5‐F introducer (Edwards LifeSciences, Irvine, USA). Under fluoroscopy guidance, a 5‐F High Fidelity Pressure catheter (Transonic® System Inc., New‐York, USA) was inserted in the aortic arch through the femoral artery introducer. A balloon‐tipped thermodilution pulmonary artery catheter (Swan‐Ganz®, Edwards LifeSciences, Irvine, USA) was inserted through the right jugular introducer and advanced until pulmonary artery under monitoring of pressure waveforms to continuously measure pulmonary arterial pressure (PAP) and cardiac output (CO) (Vigilance®, Edwards LifeSciences, Irvine, USA). A midline laparotomy was then performed to introduce a 14‐F Foley catheter (Beiersdorf AG, Hamburg, Germany) in the bladder and connect to urine bag.

#### 2.1.4 Cardiac Instrumentation

After an intravenous bolus of heparin (150 UI/kg) followed by a continuous infusion (100UI/kg/hour) to prevent thrombus formation, a 5‐F left Judkins® guiding catheter (Medtronic Inc, Minneapolis, USA) was inserted through the right carotid artery introducer in the ostium of the left coronary artery. Thereafter, a 0.014‐inch pressure‐flow sensor‐tipped guidewire (ComboWire®, Volcano Corporation, San Diego, CA, USA) was advanced, under fluoroscopy guidance, through the guiding catheter and placed with its sensor in the mid portion of the left anterior descending coronary artery (LAD). A dilatation balloon (Trek®, Abbott Vascular, Santa Clara, USA) was then guided in the proximal part of the LAD. Correct Combowire® and balloon position were confirmed by fluoroscopy after injection of angiographic contrast.

### 2.2 Experimental protocol

After instrumentation and surgical preparation, the animals were allowed to stabilize for a period of 2 hr before baseline measurements of heart rate (HR), mean arterial pressure (MAP), PAP, CO, arterial O_2_ saturation (SaO_2_) and hematocrit. Coronary pressure and flow were then subsequently measured in baseline conditions, during hyperemia achieved first with adenosine then with bradykinin and finally during stepwise inflations of the intracoronary balloon to generate a flow‐pressure relationship. All measurements were repeated after a 6‐hr period of rest.

#### 2.2.1 Coronary Pressure ‐ Flow Derived Indices

CBF reserve was defined as the ratio between hyperemic flow and baseline flow. Baseline Flow was calculated as the mean of 5 successive beats at rest. Hyperemic Flow was calculated as the mean of the three successive beats with the highest flow at maximal vasodilation. Hyperemic coronary microvascular resistance (HMR) was defined as the ratio between coronary pressure and coronary flow measured during hyperemia (24).

Hyperemia was successively obtained by intracoronary 3 ml bolus of adenosine (90µg) and 3 ml bolus of bradykinin (10^‐6^M). Three representative measurements were performed for each drug. Between each consecutive measurement, a 3 ml saline bolus was given to eliminate any residual drug in the catheter and enough time was respected to insure the return to baseline.

#### 2.2.2 Coronary Pressure ‐ Flow relationship

Coronary pressure and flow were continuously recorded during progressive and careful inflation of the intracoronary balloon until precipitous fall of flow, where the intracoronary balloon was then deflated to ovoid onset of ischemia.

The coronary autoregulatory curve was constructed using the following approach. Coronary pressure and flow coordinates were averaged at pressure steps of 5 mmHg. With the resulting pressure‐flow points, paired sets of linear regressions and their coefficient of determination R‐squared (R^2^) were calculated starting at the highest and at the lowest pressure. The lower pressure limit of autoregulation, or autoregulation breakpoint, was determined by the intersection of the two best‐fitted regression lines.

Systemic and coronary hemodynamic variables were recorded and analyzed using the Notocord‐hem Evolution® Software (NOTOCORD Systems SAS, Le Pecq, France).

## 3 RESULTS

General hemodynamics remained stable during all the protocol. HR was 85 ± 10 and 91 ± 23 bpm; MAP was 89 ± 8 and 77 ± 7 mmHg; PAP was 23 ± 3 and 23 ± 2 mmHg; CO was 4.2 ± 0.6 and 4.3 ± 1.4 L/min; SaO2 was 100 ± 0 and 99 ± 1%; hematocrit was 26.5 ± 0.9 and 24.4 ± 1.7, respectively for baseline and after 6‐hr measurements.

### 3.1 Coronary pressure ‐ flow derived indices

**Figure 1:**
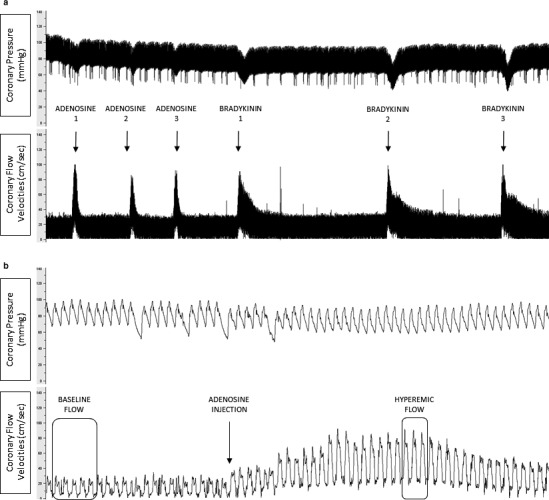
Representative measurements of coronary pressure – flow velocities derived – indices in one representative pig. (a) Coronary pressure and flow velocities evolution after pharmacologic vasodilation. Both adenosine and bradykinin tripled CBF. (b) Baseline flow was determined on a five‐beat measurement. Hyperemic flow was calculated on a three‐beat measurement during the peak of hyperemia after the vasodilator administration

Figure 1 shows coronary pressure and flow evolution after intracoronary vasodilators administration for one representative pig. Both adenosine and bradykinin approximately tripled CBF. Coronary flow, pressure and their derived indices remained stable within measurements and are summarized in Table 1.

**Table 1:** ‐ Coronary pressure and flow derived indices

Variables	ADENOSINE		BRADYKININ	
	Baseline	6 hr	Baseline	6 hr
Baseline Flow (cm/sec)	19.1 ± 3.7	20.2 ± 3.1	19.1 ± 3.5	19.3 ± 2.3
Hyperemic Flow (cm/sec)	60.3 ± 5.5	57.6 ± 3.3	59.7 ± 6.3	55.9 ± 4.4
Coronary Pressure (mmHg)	61 ± 6	71 ± 5	61 ± 4	61 ± 3
CBF reserve	3.3 ± 0.6	2.9 ± 0.2	3.2 ± 0.5	2.9 ± 0.2
HMR (mmHg/cm/sec)	1.2 ± 0.1	1.2 ± 0.1	1.2 ± 0.1	1.2 ± 0.1

Note

For each pig, data were calculated as the mean of three consecutive representative measurements. Results are expressed as mean ± *SD*. Coronary Pressure was measured during peak of hyperemia. CBF Reserve = Hyperemic Flow/ Baseline Flow. HMR = Coronary Pressure/ Hyperemic Flow measured in maximal dilation. CBF = Coronary Blood Flow; HMR = Hyperaemic Microvascular Resistance.

### 3.2 Coronary pressure ‐ flow relationship

**Figure 2:**
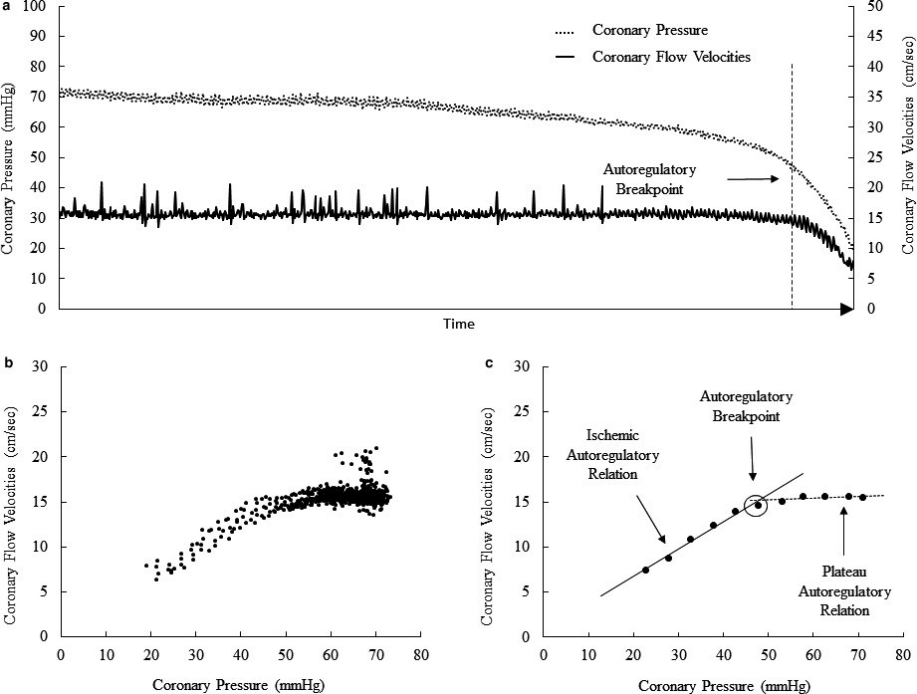
Coronary pressure ‐ flow velocities relationship for autoregulation assessment. (a) Overtime evolution in one representative pig, beat per beat, of coronary pressure (dashed line) and coronary flow velocities (solid line) following inflation of the intracoronary balloon. (b) Set of all data points recorded. (c) Individual data of panel b were pooled and averaged in increments of 5 mm Hg. Paired sets of linear regressions and their coefficient of determination R‐squared (R^2^) were then calculated. The two linear regressions presenting the best R^2^ determine the plateau (dashed line) and the ischemic autoregulatory relationship (solid line). The intersection between these two best‐fitted regression lines defines the autoregulatory break point (arrow)

Figure 2 shows the evolution of coronary pressure and flow velocities during progressive inflation of the intracoronary balloon occluder and the subsequent construction and analyze of the autoregulation curve and its autoregulatory breakpoint. The lower limit of autoregulation was consistent within animals, with individual values between 42 and 55 mmHg, and remained stable within measurements, with calculated autoregulatory breakpoint of 49 ± 5 mmHg and 49 ± 5 mmHg, respectively, at baseline and after 6 hr measurement.

## 4 DISCUSSION

The present results show the feasibility of a comprehensive method for coronary blood flow regulation assessment in an intact large animal experimental model. To our knowledge, this is the first report of concomitant coronary metabolic regulation and autoregulation including their associated microvascular and endothelial function measured altogether in the same experimental preparation.

CBF was estimated using a commercially available high‐fidelity pressure catheter equipped with an ultrasonic probe licensed for human studies. It was assumed that velocity and blood flow changes were proportional, as most of vasomotor tone‐dependent diameter changes would occur in the micro‐circulation downstream to the catheter tip. However, this may not always be true, and therefore is a limitation to our conclusions. Another obvious limitation was the small number of only four animals. However, the results were consistent and additional experiments therefore were not approved for further validation of the experimental model.

To calculate CBF reserve, maximal vasodilation was achieved pharmacologically by intracoronary administration of vasodilators. Adenosine is known to produce a merely microvascular vasodilation (with no noticeable effect on epicardial vessels) and to act directly on the vascular smooth muscle (i.e. independently of the endothelial function) (Kern et al., 2006). Bradykinin produces an endothelial‐dependent vasodilation and acts on both macro and microcirculation. In this model, we chose to administer adenosine and bradykinin by intracoronary bolus instead of steady‐state intravenous infusion in order to reduce the systemic effect of these vasodilators. In clinical practice, CBF reserve is considered as normal when above 3.0 and as pathological when it drops below the threshold value of 2.0 (Baumgart et al., 1998; Kern et al., 2006). As CBF reserve may be affected by baseline flow and by coronary perfusion pressure, coronary vascular resistance calculated during maximal vasodilatation, or HMR, has been proposed as a more reliable index of the functional state of the microcirculation (Kern et al., 2006; Meuwissen et al., 2001). There is no clearly defined cut‐off for HMR. The measurement has been previously reported in a porcine myocardial infarction model (Koudstaal et al., 2013). Consistent with the literature, CBF approximately tripled in all pigs with either adenosine or bradykinin, and HMR was not different indicating, respectively, preserved coronary microvascular and endothelial function. The coronary pressure drop during the peak of hyperemia was higher after administration of bradykinin. This may be explained by its action on all compartments of the coronary circulation contrary with adenosine which act essentially on the microcirculation.

Coronary autoregulation maintains CBF constant over a wide range of perfusion pressures from 60 to 120 mmHg depending on the species studied (Canty, 1988; Dole, 1987; Duncker et al., 2015; Feigl et al., 1990; Hoffman & Spaan, 1990). Coronary pressure‐flow relationships are described by a sigmoid curve. There is a threshold of pressure, the "autoregulatory breakpoint", under which autoregulation is compromised, where microvascular resistances are minimal and CBF becomes directly pressure‐dependent, corresponding to the “ischemic relation” part of the autoregulation curve (Figure 2). Indeed, an additional pressure drop below this critical threshold results in ischemia (Duncker et al., 2015; Hoffman & Spaan, 1990). Autoregulation of CBF has been previously studied on isolated arteries or heart preparations (Bell, Mocanu, & Yellon, 2011), and in intact animals with coronary circulation cannulated and perfused separately from the aorta by an extracorporeal pump (Mosher, Ross, McFate, & Shaw, 1964). A disadvantage of extracorporeal circulation to control CBF is loss of the vasodilatory effects of pulsatile flow (Goto, VanBavel, Giezeman, & Spaan, 1996). A more natural unanesthetized dog model has been reported in which coronary perfusion pressure was varied by an external hydraulic occluder and CBF measured either by a transit time ultrasonic flow probe (Smith & Canty, 1993), both placed around the proximal portion of the circumflex coronary artery, or by injection of radioactive microspheres (Canty, 1988). We were not able to reproduce this method as external occlusion of the left descending coronary artery in pigs resulting in a vasospasm and a sharp decrease in CBF. On the other hand, we used intracoronary velocity to estimate flow as the ultrasonic flow probe needs tight adjustment around the coronary artery, which may affect high flows but be disturbed a decreased flow. The closed‐chest model offers several additional benefits. Contrary with open‐chest model, this endovascular approach maintains local and general homeostasis and limits the variables (inflammatory response, hypothermia, intrathoracic pressures variation, drop in blood pressure, and cardiac output…) that may introduce bias. In addition, this minimally invasive technique using intracoronary wire and balloon has also the advantages of having a short preparation time and a rapid learning, of being reproducible and of preserving the native coronary vascularization including its pulsatility. In the present experiments, the calculated autoregulatory breakpoints — between 42–55 mmHg — were consistent with those previously reported (Canty, 1988; Dole, 1987; Duncker et al., 2015; Feigl et al., 1990; Hoffman & Spaan, 1990) and remained stable within measurements.

In summary, we developed, in a large intact animal model, a new method allowing integrated CBF metabolic regulation and autoregulation assessments. This new approach may be implemented for further pharmacological preclinical studies on coronary circulation, allowing translation of the results.

## 5 COMPETING INTEREST

The authors declare no conflict of interest.

## AUTHOR CONTRIBUTIONS

CB, AH, SB conceived and designed experiments; CB, FS performed experiments; CB analyzed the data; CB draft the manuscript and prepared figures; RN, JC, SB, LD, LV, and LH helped for interpretation of data, provided constructive feedback and revised the manuscript. All authors approved the final version of the paper. All authors agree to be accountable for all aspects of the work. We confirm that all persons designated as authors qualify for authorship, and all those who qualify for authorship are listed.

## References

[phy214510-bib-0001] Baumgart, D. , Haude, M. , Liu, F. , Ge, J. , Goerge, G. , & Erbel, R. (1998). Current concepts of coronary flow reserve for clinical decision making during cardiac catheterization. American Heart Journal, 136(1), 136–149. 10.1016/S0002-8703(98)70194-2 9665231

[phy214510-bib-0002] Bell, R. M. , Mocanu, M. M. , & Yellon, D. M. (2011). Retrograde heart perfusion: The Langendorff technique of isolated heart perfusion. Journal of Molecular Cellar Cardiology, 50(6), 940–950. 10.1016/j.yjmcc.2011.02.018 21385587

[phy214510-bib-0003] Canty, J. M. (1988). Coronary pressure‐function and steady‐state pressure‐flow relations during autoregulation in the unanesthetized dog. Circulation Research, 63(4), 821–836. 10.1161/01.RES.63.4.821 3168181

[phy214510-bib-0004] Chilian, W. M. (1997). Coronary microcirculation in health and disease: Summary of an NHLBI workshop. Circulation, 95(2), 522–528. 10.1161/01.CIR.95.2.522 9008472 PMC4037233

[phy214510-bib-0005] Dole, W. P. (1987). Autoregulation of the coronary circulation. Progress in Cardiovascular Diseases, 29(4), 293–323. 10.1016/S0033-0620(87)80005-1 3809516

[phy214510-bib-0007] Duncker, D. J. , Koller, A. , Merkus, D. , & Canty, J. M. (2015). Regulation of coronary blood flow in health and ischemic heart disease. Progress in Cardiovascular Diseases, 57(5), 409–422. 10.1016/j.pcad.2014.12.002 25475073 PMC5856234

[phy214510-bib-0008] Durand, M. J. , & Gutterman, D. D. (2013). Diversity in mechanisms of endothelium‐dependent vasodilation in health and disease. Microcirculation, 20(3), 239–247. 10.1111/micc.12040 23311975 PMC3625248

[phy214510-bib-0009] Feigl, E. O. , Neat, G. W. , & Huang, A. H. (1990). Interrelations between coronary artery pressure, myocardial metabolism and coronary blood flow. Journal of Molecular and Cellular Cardiology, 22(4), 375–390. 10.1016/0022-2828(90)91474-L 2388275

[phy214510-bib-0010] Furchgott, R. F. , & Zawadzki, J. V. (1980). The obligatory role of endothelial cells in the relaxation of arterial smooth muscle by acetylcholine. Nature, 288, 373–376. 10.1038/288373a0 6253831

[phy214510-bib-0011] Goto, M. , VanBavel, E. , Giezeman, M. J. , & Spaan, J. A. (1996). Vasodilatory effect of pulsatile pressure on coronary resistance vessels. Circulation Research, 79(5), 1039–1045. 10.1161/01.RES.79.5.1039 8888697

[phy214510-bib-0012] Hoffman, J. I. , & Spaan, J. A. (1990). Pressure‐flow relations in coronary circulation. Physiological Reviews, 70(2), 331–390. 10.1152/physrev.1990.70.2.331 2181499

[phy214510-bib-0013] Kern, M. J. , Lerman, A. , Bech, J. W. , De Bruyne, B. , Eeckhout, E. , Fearon, W. F. , … Spaan, J. A. (2006). Physiological assessment of coronary artery disease in the cardiac catheterization laboratory: A scientific statement from the American Heart Association committee on diagnostic and interventional cardiac catheterization Council on Clinical Cardiology. Circulation, 114(12), 1321–1341. 10.1161/CIRCULATIONAHA.106.177276 16940193

[phy214510-bib-0014] Koudstaal, S. , Jansenof Lorkeers, S. J. , van Slochteren, F. J. , van der Spoel, T. I. G. , van de Hoef, T. P. , Sluijter, J. P. , … Chamuleau, S. A. J. (2013). Assessment of coronary microvascular resistance in the chronic infarcted pig heart. Journal of Cellular and Molecular Medecine, 17(9), 1128–1135. 10.1111/jcmm.12089 PMC4118172 23910946

[phy214510-bib-0015] Meuwissen, M. , Chamuleau, S. A. , Siebes, M. , Schotborgh, C. E. , Koch, K. T. , de Winter, R. J. , … Piek, J. J. (2001). Role of variability in microvascular resistance on fractional flow reserve and coronary blood flow velocity reserve in intermediate coronary lesions. Circulation, 103(2), 184–187. 10.1161/01.CIR.103.2.184 11208673

[phy214510-bib-0016] Mosher, P. , Ross, J. , McFate, P. A. , & Shaw, R. F. (1964). Control of coronary blood flow by an autoregulatory mechanism. Circulation Research, 14(3), 250–259. 10.1161/01.RES.14.3.250 14133952

[phy214510-bib-0017] Smith, T. P. , & Canty, J. M. (1993). Modulation of coronary autoregulatory responses by nitric oxide. Evidence for flow dependent resistance adjustments in conscious dogs. Circulation Research, 73(2), 232–240. 10.1161/01.RES.73.2.232 8330372

[phy214510-bib-0018] Tune, J. D. , Gorman, M. W. , & Feigl, E. O. (2004). Matching coronary blood flow to myocardial oxygen consumption. Journal of Applied Physiology, 97(1), 404–415. 10.1152/japplphysiol.01345.2003 15220323

